# Antibacterial effects of *Lactobacillus* and bacteriocin PLNC8 αβ on the periodontal pathogen *Porphyromonas gingivalis*

**DOI:** 10.1186/s12866-016-0810-8

**Published:** 2016-08-18

**Authors:** Hazem Khalaf, Sravya Sowdamini Nakka, Camilla Sandén, Anna Svärd, Kjell Hultenby, Nikolai Scherbak, Daniel Aili, Torbjörn Bengtsson

**Affiliations:** 1 Faculty of Medicine and Health, School of Health and Medical Sciences, Örebro University, Örebro, Sweden; 2 PEAS Institut AB, Söderleden 1, Linköping, Sweden; 3 Division of Molecular Physics, Department of Physics, Chemistry and Biology (IFM), Linköping University, Linköping, Sweden; 4 Division of Clinical Research Centre, Department of Laboratory Medicine, Karolinska Institutet, Stockholm, Sweden; 5 School of Science and Technology, Örebro University, Örebro, Sweden

**Keywords:** Periodontitis, *P. gingivalis*, *Lactobacillus*, Bacteriocin, PLNC8

## Abstract

**Background:**

The complications in healthcare systems associated with antibiotic-resistant microorganisms have resulted in an intense search for new effective antimicrobials. Attractive substances from which novel antibiotics may be developed are the bacteriocins. These naturally occurring peptides are generally considered to be safe and efficient at eliminating pathogenic bacteria. Among specific keystone pathogens in periodontitis, *Porphyromonas gingivalis* is considered to be the most important pathogen in the development and progression of chronic inflammatory disease. The aim of the present study was to investigate the antimicrobial effects of different *Lactobacillus* species and the two-peptide bacteriocin PLNC8 αβ on *P. gingivalis*.

**Results:**

Growth inhibition of *P. gingivalis* was obtained by viable *Lactobacillus* and culture media from *L. plantarum* NC8 and 44048, but not *L. brevis* 30670. The two-peptide bacteriocin from *L. plantarum* NC8 (PLNC8 αβ) was found to be efficient against *P. gingivalis* through binding followed by permeabilization of the membranes, using Surface plasmon resonance analysis and DNA staining with Sytox Green. Liposomal systems were acquired to verify membrane permeabilization by PLNC8 αβ. The antimicrobial activity of PLNC8 αβ was found to be rapid (1 min) and visualized by TEM to cause cellular distortion through detachment of the outer membrane and bacterial lysis.

**Conclusion:**

Soluble or immobilized PLNC8 αβ bacteriocins may be used to prevent *P. gingivalis* colonization and subsequent pathogenicity, and thus supplement the host immune system against invading pathogens associated with periodontitis.

**Electronic supplementary material:**

The online version of this article (doi:10.1186/s12866-016-0810-8) contains supplementary material, which is available to authorized users.

## Background

There is today an intense search for new antimicrobials with effective activity and with less possibility to induce antimicrobial resistance [1]. Attractive substances from which novel antibiotics may be developed are the bacteriocins, a group of bacterially produced compounds used to fight other bacteria [2]. Bacteriocins have a net positive charge and express amphipathic structures and kill microbes through interaction with negatively charged microbial membrane structures, such as Lipopolysaccharide and Lipoteichoic Acid. These mechanisms are more difficult to evade by developing resistance, compared to metabolic enzymes which usually are targets for conventional antibiotics.

Periodontitis is a common chronic inflammatory disease caused by an accumulation of different pathogenic biofilm-forming bacteria in dental pockets, leading to an exaggerated immune response that destructs periodontal ligament and causes alveolar bone loss. *P. gingivalis* is considered a keystone pathogen among these pathogenic bacteria and is well associated with the development of periodontitis. *P. gingivalis* is an anaerobic gram-negative, black-pigmented bacterium that has been widely associated as a putative organism in causing aggressive periodontitis and progression into systemic diseases [3]. The ability of *P. gingivalis* to invade host cells, including gingival epithelial cells [4] and heart and aortic endothelial cells [5] demonstrate a possible mechanism for its establishment and subsequent pathogenesis by evading the host immune system. *P. gingivalis* express multiple virulence factors (fimbriae, capsule, LPS, proteinases and toxic end-products) that contribute significantly to their pathogenicity by altering host immune responses [6]. However, *P. gingivalis* virulence is primarily associated with the elevated proteolytic activity of cysteine proteinases (reviewed in [7]) that are divided into arginine-specific (Rgp) and lysine-specific (Kgp) [8]. Besides the immunomodulatory effects of *P. gingivalis*, the bacterium is also able to form biofilms containing extracellular polymeric substances that provides antibiotic resistance and a niche for long-term survival within the host by evading host immune responses. These biofilms are difficult to treat and conventional methods are still being used to treat patient with periodontitis, including mechanical removal of hard and soft subgingival biofilms, scaling and chemical treatment to control plaque (metallic salts, antibiotics and phenols). These methods are less efficient since within a few hours new biofilms are formed that are prone to cause infections [9]. Furthermore, periodontitis has been associated with other systemic conditions, such as atherosclerosis, following identification of periodontal pathogens, including *P. gingivalis*, in atherosclerotic plaques [3, 10]. We have recently shown that *P. gingivalis* induces gene expression of angiopoietin 2 in aortic smooth muscle cells, which increases the migration of the cells that is associated with the pathogenesis of atherosclerosis [11].

The oral cavity harbours a wide variety of bacteria from the genus *Lactobacillus* that play an important role in the maintenance of a healthy balance of the oral ecosystem and are suggested to have protective effects in the pathogenesis of periodontal disease [12, 13]. However, many *Lactobacillus* species are outcompeted by gram-negative pathogens, including *P. gingivalis*, during disease progression. It has previously been reported that certain *Lactobacillus* species, including *L. rhamnosus* and *L. salivarius*, possess antimicrobial activity against periodontal pathogens, such as *P. gingivalis* [14]. Pangsomboon and colleagues [15] reported that bacteriocins from *L. paracasei* were able to kill *P. gingivalis* at a minimal concentration of 0.14 mM. Bacteriocins are divided into three classes depending on their characteristics. Class I (lantibiotics) are small peptides (<5 kDa) that contain unusual amino acids, lanthionine and three-methyllanthionine, introduced by post-translational modifications. Class II bacteriocins are synthesized in precursor forms that are processed after two glycine residues and display structural stability against proteolysis, heat and a wide range of pH. This group also includes bacteriocins composed of two different peptides that dimerize to form an active poration complex. Class III includes bacteriocins with large molecules that are sensitive to heat. Bacteriocins are antimicrobial compounds secreted by bacteria as part of their defence mechanism. Likewise, *L. plantarum* NC8 has previously been shown to produce a two-peptide bacteriocin, composed of PLNC8 α and β, which is classified as a class II bacteriocin. PLNC8 αβ are heat-stable and reported to possess antimicrobial activity towards gram-positive bacteria [16].

It is important to determine the anti-bacterial activity of *Lactobacillus* species and their bacteriocins, as an alternative method to antibiotic therapy, in preventing the colonization of *P. gingivalis*. We hypothesize that specific *Lactobacillus* species are able to suppress *P. gingivalis* growth, which is primarily due to expression and secretion of bacteriocins, and that these substances may be successful in the prevention of periodontitis. The aim of the present study was to elucidate the properties and antimicrobial effects of different *Lactobacillus* species and the two-peptide bacteriocin PLNC8 αβ on *P. gingivalis*.

## Methods

### Bacterial culture conditions

*P. gingivalis* wild type strains ATCC 33277 (ATCC, Manassas, VA) and W50, and the W50-derived Kgp proteinase and Rgp proteinase mutant strains (K1A and E8, respectively) were a kind gift from Dr. M.A. Curtis (Molecular Pathogenesis Group, Queen Mary, University of London). *P. gingivalis* strains were grown in anaerobic conditions (80 % N_2_, 10 % CO_2_, and 10 % H_2_) at 37 °C in an anaerobic chamber (Concept 400 Anaerobic Workstation; Ruskinn Technology Ltd., Leeds, United Kingdom). The bacterial concentration was adjusted to correlate with approximately 10^9^ CFU/ml, which was determined by viable count by culturing the bacteria on fastidious anaerobe agar (45.7 g/l, pH 7.2, Acumedia, Neogen, Lansing, USA), supplemented with 5 % defibrinated horse blood for 5 days.

The *Lactobacillus* strains *L. plantarum* NC8, *L. plantarum* 44048 and *L. brevis* 30670 (Culture Collection, University of Gothenburg, Sweden) were grown on deMan Rogosa Sharp (MRS, BD Science) supplemented with agar (Difco) at 37 °C for 24 h in a jar containing an oxygen-free environment (Anaerobic pouch system EZ, BD Biosciences, CA, USA). Fresh cultures were used to inoculate MRS broth (Difco, BD Biosciences, CA, USA), and grown statically and anaerobically for 24 h at 37 °C. *Lactobacillus* were grown from a 0.5 % inoculum for 24 h at 37 °C under anaerobic and static growth conditions, and were then used for further experiments. The two-peptide bacteriocin from *L. plantarum* NC8, PLNC8 α and β, were purchased from GL Biochem (Shanghai) Ltd, China. Amino acid sequences for the peptides are:

- PLNC8α- DLTTKLWSSWGYYLGKKARWNLKHPYVQF and
- PLNC8β- SVPTSVYTLGIKILWSAYKHRKTIEKSFNKGFYH [16]

### Liposome preparation

Liposomes were prepared according to methods that are well established in the field [17, 18]. Briefly, liposomes were prepared by dry film formation, hydration and finally extrusion through a polycarbonate membrane to form monodisperse large unilamellar vesicles. The lipids 1-palmitoyl-2-oleoyl-sn-glycero-3-phospho-L-serine (POPS) and 1-palmitoyl-2-oleoyl-sn-glycero-3-phosphatidylcholine (POPC) (Avanti Polar Lipids, Alabaster, USA) were mixed at molar ratios 1:99, 5:95 and 10:90 while dissolved in chloroform. A dry lipid film was formed by evaporation of the chloroform by nitrogen flow and overnight lyophilization. The film was hydrated with either 10 mM phosphate buffer (PB) pH 7 or 10 mM phosphate buffer saline (PBS) pH 7, and the solution was vortexed for 1 min and put on a shaker for 1 h before extruded 21 times through a 100 nm pore-sized polycarbonate membrane. For fluorescence leakage assay the lipid film was hydrated with buffer (PBS) containing self-quenching concentration (50 mM) of 5 (6)-carboxyfluorescein (CF) (Sigma Aldrich) and liposomes were prepared as described above. Removal of unencapsulated CF was done by gel filtration using a PD-25 column (GE Healthcare, Uppsala, Sweden) and liposomes with encapsulated CF were eluted with PBS.

### Dynamic light scattering and Zeta potential

In order to mimic *P. gingivalis* membrane composition and potential, the hydrodynamic radius and zeta potential was measured on liposomes suspended in 10 mM PB pH 7 and suspensions containing microvesicles from *P. gingivalis* W50, using a Malvern ZetaSizer Nano S (Malvern Instruments Ltd, UK) and a disposable cuvette.

### Carboxyfluorescein (CF) release assay

Leakage of the liposome encapsulated fluorophore CF due to additions of the bacteriocins was recorded using a fluorescence plate reader (Infinite 200, Tecan, Austria) where λ_ex_ = 485 nm and λ_em_ = 520 nm. CF was encapsulated at self-quenching concentration, and CF release results in an increased fluorescence signal. Liposomes were diluted to 25 μM (total lipid concentration) in PBS, followed by additions of 0, 0.005, 0.01, 0.02, 0.05, 0.1, 0.2, 0.5, 1 and 2 μM of the peptides PLNC8 α and β, separately and combined. In order to estimate the maximum release from each sample, a final addition of 0.5 % Triton X-100 was made at the end of all measurements and the total amount of CF (100 % release) was estimated after 15 min incubation. The CF release is presented as percentage release for each time interval (measurements taken every minute). The percentage CF release is calculated as 100 × (F – F_0_)/(F_T_ – F_0_) where F_0_ is the initial fluorescence intensity of CF before peptide addition, F is the fluorescence intensity of CF at time point *t* and F_T_ is the maximum fluorescence after the addition of Triton X-100.

### Circular Dichroism (CD) spectroscopy

Bacteriocins are often unstructured in solution but typically adopt a well-defined secondary structure when bound to the bacterial cell membrane as a result of membrane partitioning [19]. The secondary structure of the bacteriocins was investigated using CD spectroscopy. CD spectra were recorded using a Chirascan spectropolarimeter (Applied Photophysics, UK) and a 1 mm quartz cuvette at 20 °C with a sampling interval of 0.5 nm. All measurements were done in triplicates and averaged before converted to mean residue ellipticity (MRE) and curves smoothened using Savitzky-Golay algorithm.

### Antimicrobial activity of Lactobacillus

The ability of different *Lactobacillus* strains to inhibit *P. gingivalis* growth was assessed on fastidious anaerobe agar plates. Briefly, different *P. gingivalis* strains (10^8^ CFU in 100 μl) were spread onto fastidious anaerobe agar plates and allowed to dry. *Lactobacillus* were diluted in MRS broth and 10 μl drops (10^6^ CFU) were placed onto the *P. gingivalis* layer. The plates were incubated for 4 days, after which images were acquired with Olympus SZX9 at 10× magnification and the zone of inhibition was measured using the software ImageJ.

Antimicrobial activity was also assessed using *Lactobacillus* culture media (MRS broth), in which different *Lactobacillus* strains were cultured for 24 h. The bacteria were removed by centrifugation at 7000 × g and the supernatants were sterile filtered (0.2 μm). The pH was measured and the supernatants were used to determine antimicrobial activity against *P. gingivalis* on fastidious anaerobe agar plates (10 μl drops), as mentioned above.

### Antimicrobial activity of bacteriocin PLNC8 αβ

The antimicrobial activity of PLNC8 αβ on *P. gingivalis* was visualized using the fluorescent dye Sytox® Green, which can only cross damaged membranes and fluoresce upon binding to nucleic acids. *P. gingivalis* were washed and resuspended in Krebs-Ringer Glucose buffer (KRG) (120 mM NaCl, 4.9 mM KCl, 1.2 mM MgSO_4_, 1.7 mM KH_2_PO_4_, 8.3 mM Na_2_HPO_4_, and 10 mM glucose, pH 7.3) and incubated in the presence or absence of PLNC8 αβ in 96-well microtiter plates. Images were captured with Olympus BX41 at 40× magnification.

Transmission electron microscopy (TEM) was used to visualize the damage of *P. gingivalis*, caused by PLNC8 αβ. Briefly, *P. gingivalis* ATCC 33277 were pelleted and washed with KRG. The bacteria were then treated with 280 nM of PLNC8 αβ in a molar ratio of 1:2 for 2 min and 10 min, followed by fixation in 2.5 % glutaraldehyde in 0.1 M phosphate buffer, pH 7.3. Specimens were washed in 0.1 M phosphate buffer, postfixed in 2 % osmium tetroxide in 0.1 M phosphate buffer for 2 h and embedded into LX-112 (Ladd, Burlington, Vermont, USA). Ultrathin sections (approximately 50-60 nm) were cut by a Leica ultracut UCT/ Leica EM UC 6 (Leica, Wien, Austria). Sections were contrasted with uranyl acetate followed by lead citrate and examined in a Hitachi HT 7700 (Tokyo, Japan). Digital images were taken by using a Veleta camera (Olympus Soft Imaging Solutions, GmbH, Münster, Germany).

### SPR analysis

Surface plasmon resonance analysis was performed by immobilizing PLNC8 αβ peptides in 1:2 molar ratio onto a carboxymethylated dextran (CM-5 sensor chip, GE-Healthcare GmbH, Uppsala, Sweden) using biacore 2000 instrument equipped with four flow cells (GE-Healthcare GmbH, Uppsala, Sweden). Each channel of the chip was immobilized with 3 different concentrations of PLNC8 αβ (2.8, 28, 280 nM) respectively, and the fourth channel was used as a blank for negative reference subtraction between the channels. HBS-EP (0.01 M HEPES, pH7.4, 0.15 M NaCl, 3 mM EDTA, 0.005 % surfactant P20) (GE-Healthcare GmbH) was used as running buffer and the flow cell temperature was set to 25 °C in all experiments. Immobilization was a 3-step process performed using amine coupling kit (GE-Healthcare GmbH, Uppsala, Sweden) where the chip surface was activated using 200 mM N-ethyl-N0-(3 diethylaminopropyl) carbodimide (EDC) and 50 mM N-hydroxysuccinimide (NHS) mixture. PLNC8 αβ peptides diluted in acetate 4.5 buffer (GE-Healthcare GmbH, Uppsala, Sweden) were immobilized to the activated surface. Ethanolamine-HCl (pH 8.5) was used to deactivate the surface to enable an efficient binding of samples to the immobilized ligand. The contact time was 7 min, which resulted in immobilization levels between 1350 and 1750 response units (RU). One thousand RU corresponds to a surface peptide concentration of about 1 ng/mm^2^.

*P. gingivalis* were washed and prepared as described above after which the bacteria were pre-incubated with 2.8, 28 or 280 nM of PLNC8 αβ for 5 min at room temperature before analysis. *P. gingivalis* without PLNC8 αβ was used as positive control. The binding affinity of the bacteria to the peptides immobilized on each channel of the chip was measured in response units (RU). 1RU = 1 pg/mm^2^ using Bia-evaluation software [20].

Immobilization of surfaces with *P. gingivalis*-specific antibodies produced and tested as described in our previous studies [21], were used to study the binding affinity of *P. gingivalis* with and without PLNC8 αβ to the immobilized antibodies. Samples were prepared as described above and the responses obtained from Bia-evaluation software were plotted. The immobilization response for anti-*P. gingivalis* antibodies was 6400 RU.

### Statistical analysis

All data were analyzed using GraphPad Prism 5.0 (GraphPad Software, La Jolla, CA, USA). One-way ANOVA with Tukey’s multiple comparison test was used for the comparisons between the different treatments. *P*-values are referred to as *,#*p* < 0.05; **,##*p* < 0.01; ***,###*p* < 0.001.

## Results

### Lactobacillus spp. suppress P. gingivalis growth

**Fig. 1:**
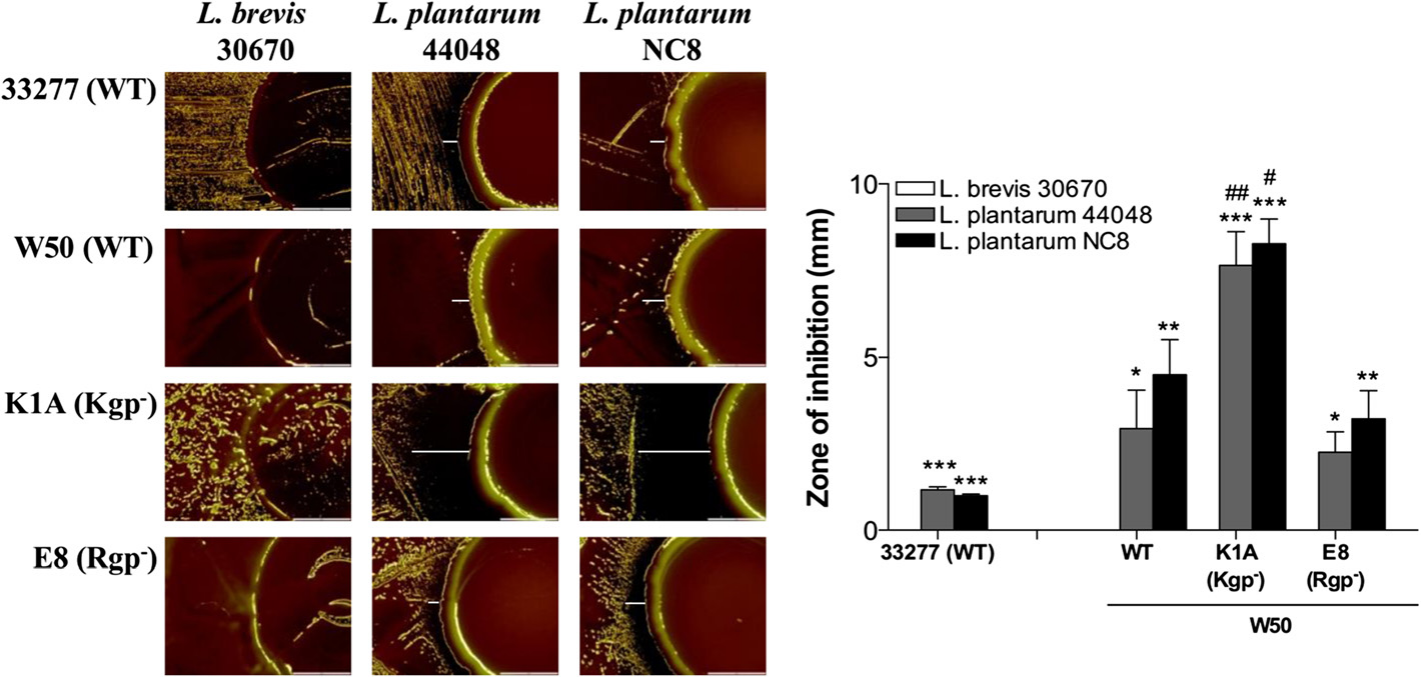
*Lactobacillus* suppresses *P. gingivalis* growth. Images were acquired after coculture of *P. gingivalis* with *Lactobacillus* for 4 days, using Olympus SZX9 at 10× magnification. The zone of inhibition was measured using ImageJ, *n* = 3. White lines indicate the inhibition zone. Prevention of *P. gingivalis* growth by *Lactobacillus* was shown to be species dependent. */#*p* < 0.05; **/##*p* < 0.01; ***/###*p* < 0.001, *- significance from *L. brevis* 30670 within each *P. gingivalis* strain, #- significance from WT W50 between the same *Lactobacillus* strain

Antimicrobial activity of different *Lactobacillus* strains (*L. brevis* 30670, *L. plantarum* 44048 and *L. plantarum* NC8) was assessed by co-culture with different *P. gingivalis* strains. *L. brevis* 30670 did not affect the growth of any of the *P. gingivalis* strains, while *L. plantarum* 44048 and NC8 could significantly inhibit all *P. gingivalis* strains (Fig. 1). Interestingly, *P. gingivalis* that lack Kgp proteinase activity was more susceptible to growth inhibition than the wild-type (WT) and the Rgp-deficient strain E8.

Since *Lactobacillus* are able to lower the pH in their environment, we have investigated if the antimicrobial activity of lactic acid bacteria was due to acidic pH. The growth media from all three *Lactobacillus* strains were found to be acidic (pH 3.4-4.1). However, antimicrobial activity against *P. gingivalis* was only found in media derived from *L. plantarum* 44048 and *L. plantarum* NC8 (data not shown). This prompted us to determine if these effects are due to bacteriocins, since acidic pH was not a contributing factor for the observed antimicrobial activity.

**Fig. 2:**
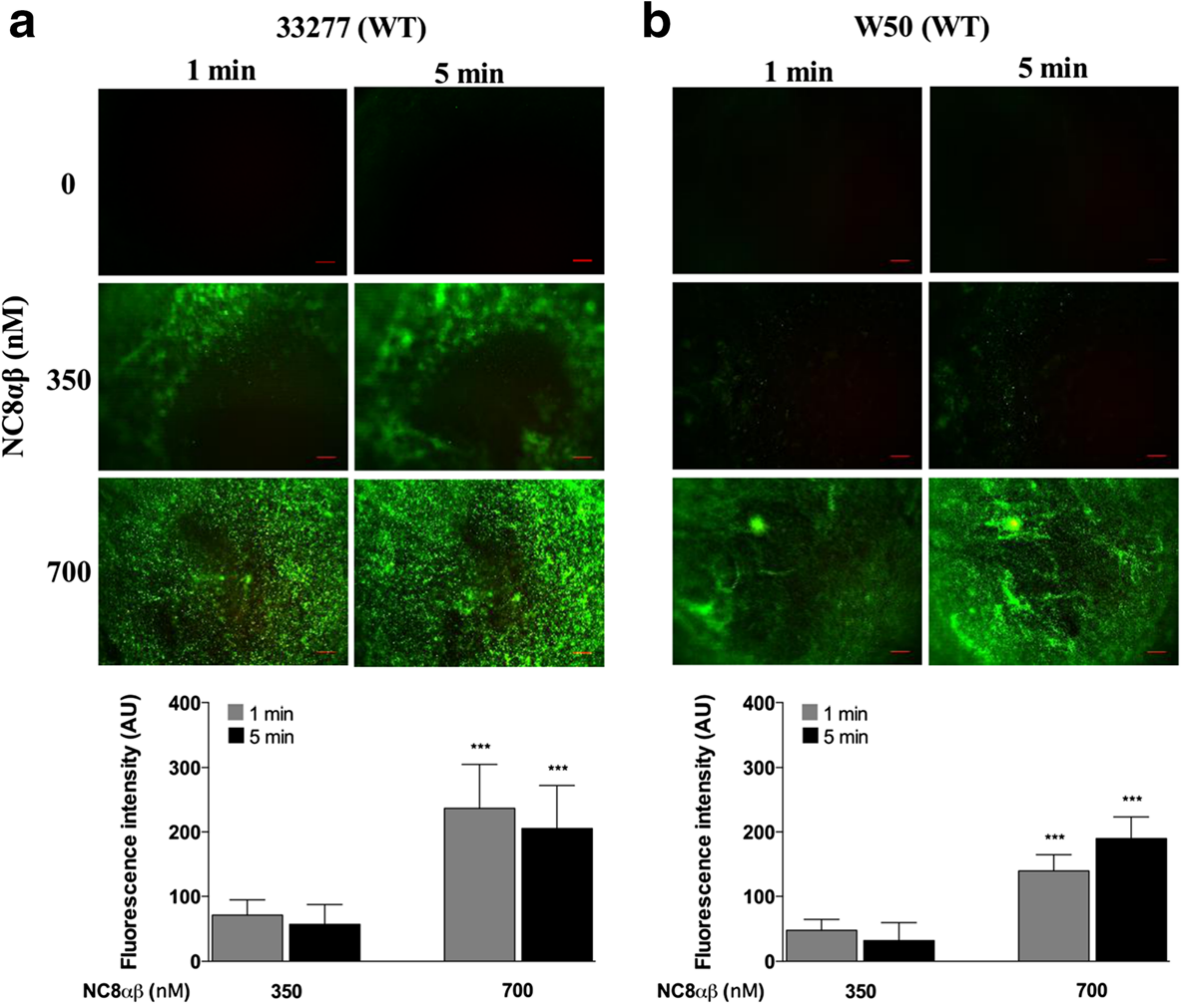
Bacteriocin PLNC8 αβ from *L. plantarum* NC8 is efficient against *P. gingivalis*. The antimicrobial activity of PLNC8 αβ on wild type (WT) *P. gingivalis* ATCC 33277 (**a**) and W50 (**b**), respectively, was visualized using the fluorescent dye Sytox® Green. Images were acquired using Olympus BX41 at 40× magnification. The antimicrobial effect of PLNC8 αβ was rapid and a significant number of *P. gingivalis* cells could fluoresce already after 1 min, indicating damaged membranes. Representative images and quantitative data of at least three independent experiments are shown. Quantitative data were normalized and the controls at each time point were set to 1. ***p < 0.001, *- significance from the control at each time point. Scale bar = 300 μm

PLNC8 α and β are two peptides produced by *L. plantarum* NC8 that form an active poration complex with antimicrobial activity. Thus, the effect of PLNC8 αβ on *P. gingivalis* viability was investigated by utilizing the fluorescent dye Sytox Green, which can only cross damaged membranes and fluoresce upon binding to nucleic acids. The uptake of Sytox Green by wild type *P. gingivalis* ATCC 33277 (Fig. 2a) and W50 (Fig. 2b) was significantly enhanced with increasing concentrations of PLNC8 αβ, which indicates a disordered integrity of *P. gingivalis* membranes. Interestingly, the effect of PLNC8 αβ bacteriocins was observed to be instant, as fluorescence was detected immediately after addition of PLNC8 αβ, reaching maximum intensity already after 1 min.

### Influence of PLNC8 αβ on model lipid membrane integrity

**Fig. 3:**
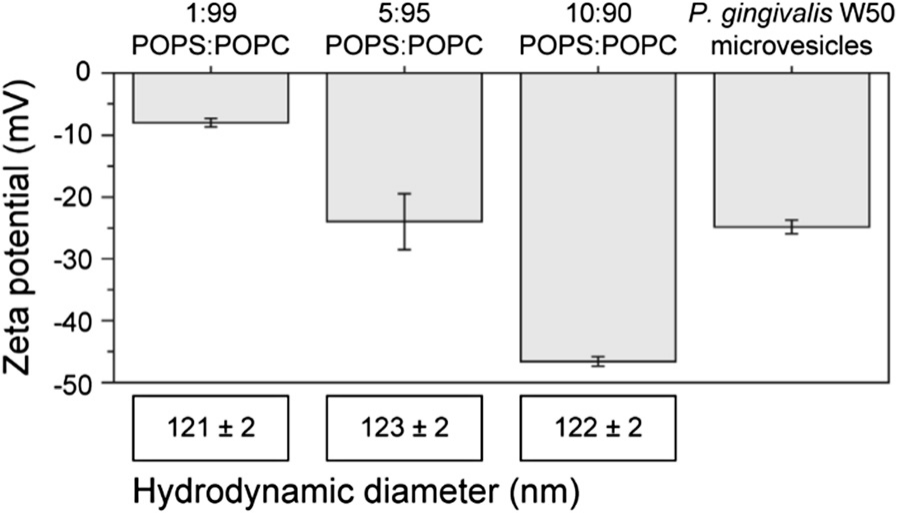
Zeta potential and size of liposomes and W50 microvesicles. The zeta potential and size of liposomes with different lipid composition was measured to identify the best match with microvesicles from *P. gingivalis* W50. All measurements are done in triplicates

A carboxyfluorescein (CF) release assay was used in order to investigate the effect of the bacteriocins on the integrity of model lipid membranes. Extrusion of liposomes resulted in large unilamellar vesicles (LUVs) with a hydrodynamic diameter of about 123 nm and a zeta potential of -24 mV. The lipid composition (5:95 POPS: POPC) was chosen to mimic the zeta potential of microvesicles present in the supernatant retrieved cultures of *P. gingivalis* W50 (-24.8 mV) (Fig. 3).

**Fig. 4:**
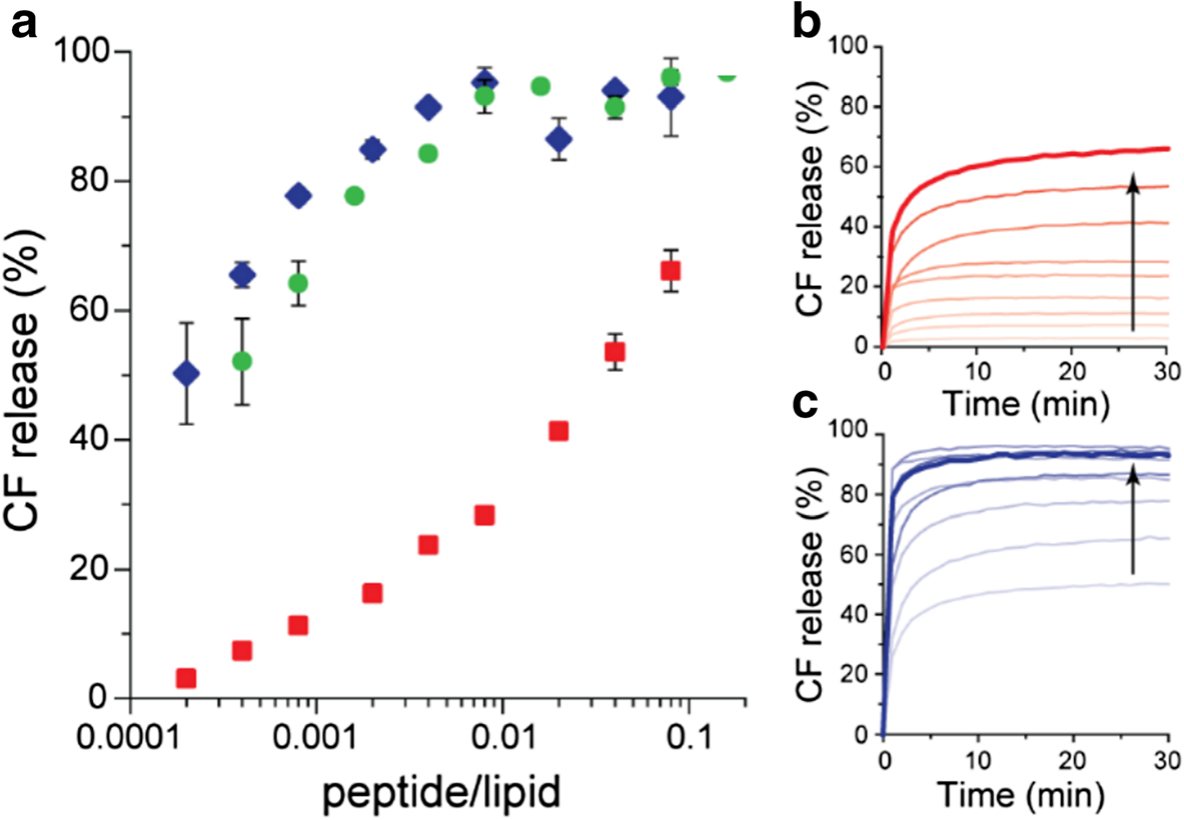
Dose- and time dependent release of CF in response to bacteriocin PLNC8 αβ. **a** Release of 5 (6)-carboxyfluorescein (CF) from liposomes (5:95 POPS: POPC) after 30 min with addition of PLNC8 α (red), β (blue) and αβ 1:2 (green). The interaction kinetics was recorded every minute for PLNC8 α (**b**) and PLNC8 β (**c**) with the liposomes and is displayed in increasing bacteriocin concentration (0.005–2 μM) indicated by the arrow. The total lipid concentration was kept constant at 25 μM. CF release without addition of bacteriocins were < 1 % and all data points are average of *n* = 3. The thick red and blue lines in B and C indicate the highest peptide concentration

The CF release was monitored over time using concentrations of bacteriocins ranging from 0.005 to 2 μM. The relative release after 30 min incubation with PLNC8 α, PLNC8 β and PLNC8 αβ (molar ratio 1:2) is shown in Fig. 4a. While CF release without addition of bacteriocins were < 1 %, a 30 min incubation with 2 μM PLNC8 α caused about 70 % release of CF and the same concentration of PLNC8 β induced almost complete release of the liposomal content (97.5 %). It is noteworthy that even for low concentrations of PLNC8 β the release of CF was high. As little as 0.05 μM (~0.002 peptide/lipid) PLNC8 β caused about 80 % CF release in 30 min. For PLNC8 αβ, the release kinetics and extent of release was similar to PLNC8 β alone for concentrations < 0.2 μM, whereas higher concentrations resulted in slightly higher release for PLNC8 αβ. The CF release assay thus indicates that both peptides bind to lipid membranes resulting in a significant reduction in membrane integrity. Interestingly, in model membranes the effect of PLNC8 β was comparable to the effect of PLNC8 αβ and the contribution from PLNC8 α thus appeared to be almost negligible. PLNC8 α alone did however cause significant leakage of CF albeit at a higher concentration than PLNC8 αβ. The release kinetics for PLNC8 α and β (Fig. 4b and c respectively) show that membrane disruption was almost immediate after exposure to the bacteriocins. Within the first couple of minutes, the CF release had already saturated. The maximum release increased with increasing concentration of bacteriocin. At lower concentrations a large fractions of liposomes were unaffected.

### Bacteriocin secondary structure

**Fig. 5:**
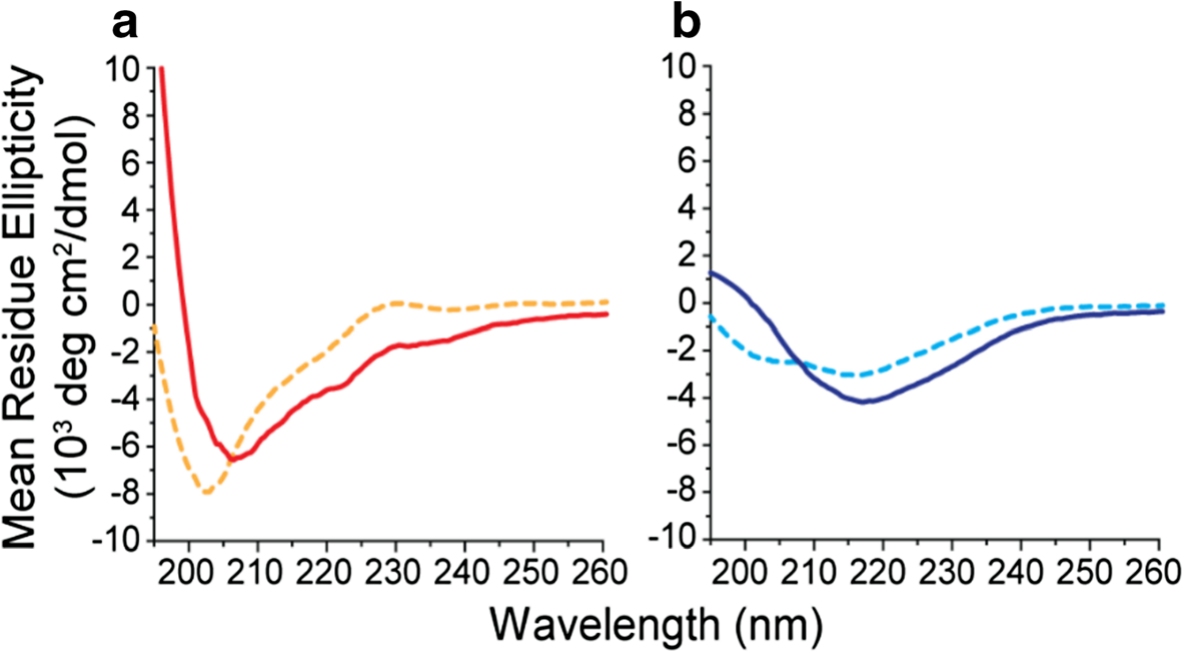
Secondary structure change of PLNC8 α and β. CD spectra of 100 μM PLNC8 α (**a**) and PLNC8 β (**b**) in solution (dashed lines) and with 1 mM (total lipid concentration) 5:95 POPS:POPC liposomes (solid lines) in 10 mM PB pH 7, 20 °C

By using CD spectroscopy we found that PLNC8 α showed no signs of ordered secondary structure (i.e. random coil) in the absence of lipid membranes (Fig. 5). PLNC8 β, on the other hand, showed CD spectra indicative of α-helical secondary structure. In the presence of POPS:POPC (5:95) LUVs, both peptides underwent a structural transition, which was quite prominent for PLNC8 β that adopted a distinct β -sheet conformation. PLNC8 α demonstrated a shift in the CD spectrum indicating a certain amount of α-helical structure.

### The bacteriocin PLNC8 αβ binds to P. gingivalis

**Fig. 6:**
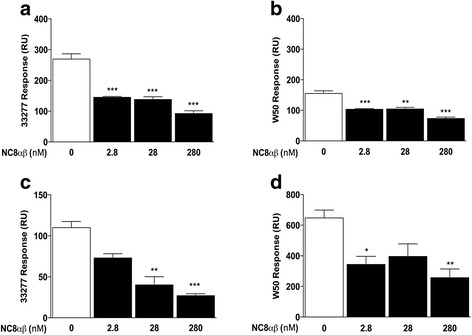
PLNC8 αβ binds to *P. gingivalis* in a dose-dependent manner. Binding of *P. gingivalis* to PLNC8 αβ and to *anti- P.gingivalis* antibodies was analyzed by SPR. Both *P. gingivalis* ATCC 33277 (**a**) and W50 (**b**) were found to bind to immobilized PLNC8 αβ (280 nM). The binding was verified by pre-incubating the bacteria with different concentrations of soluble PLNC8 αβ prior to analysis, which resulted in a significantly reduced binding to the immobilized bacteriocins. Pre-incubation of *P. gingivalis* ATCC 33277 (**c**) and W50 (**d**) with increasing concentrations of soluble PLNC8 αβ prior to analysis reduced the bacterial binding to anti-*P. gingivalis* antibodies in a dose-dependent manner. Results are presented from three independent experiments. *p < 0.05; **p < 0.01; ***p < 0.001

The antimicrobial activity of bacteriocins is dependent on binding to common epitopes on bacterial surfaces. Although binding of bacteriocins alone does not attribute for damage of bacterial membranes, we show that the antimicrobial effect of PLNC8 αβ on *P. gingivalis* is evident (Fig. 2). It is therefore important to determine if these effects are specific through binding of PLNC8 αβ to *P. gingivalis*. Both *P. gingivalis* ATCC 33277 (Fig. 6a) and W50 (Fig. 6b) were shown to bind to immobilized bacteriocin PLNC8 αβ (280 nM). Furthermore, pre-incubation of *P. gingivalis* with increasing concentrations of PLNC8 αβ caused a dose-dependent decrease in binding of the bacteria to the immobilized PLNC8 αβ peptides. Interestingly, pre-incubation of the bacteria with low concentrations of PLNC8 αβ (2.8 nM) were able to significantly decrease the binding, compared to untreated bacteria, indicating high specificity and binding affinity. Similar results were obtained with channels immobilized with 2.8, 28 and 2800 nM of PLNC8 αβ and with similar pre-treatments (data not shown).

The binding of PLNC8 αβ to *P. gingivalis* was verified using channels immobilized with anti-*P. gingivalis* antibodies. Both ATCC 33277 (Fig. 6c) and W50 (Fig. 6d) bound to the immobilized antibodies. This binding was significantly reduced in a dose-dependent manner following pre-incubation of the bacteria with increasing concentrations of PLNC8 αβ. The results suggest that the damage caused by PLNC8 αβ bacteriocins alters the integrity of *P. gingivalis* membranes, resulting in modified epitopes with reduced binding to the antibodies, compared to untreated bacteria.

### Rapid rupture of the outer membrane

**Fig. 7:**
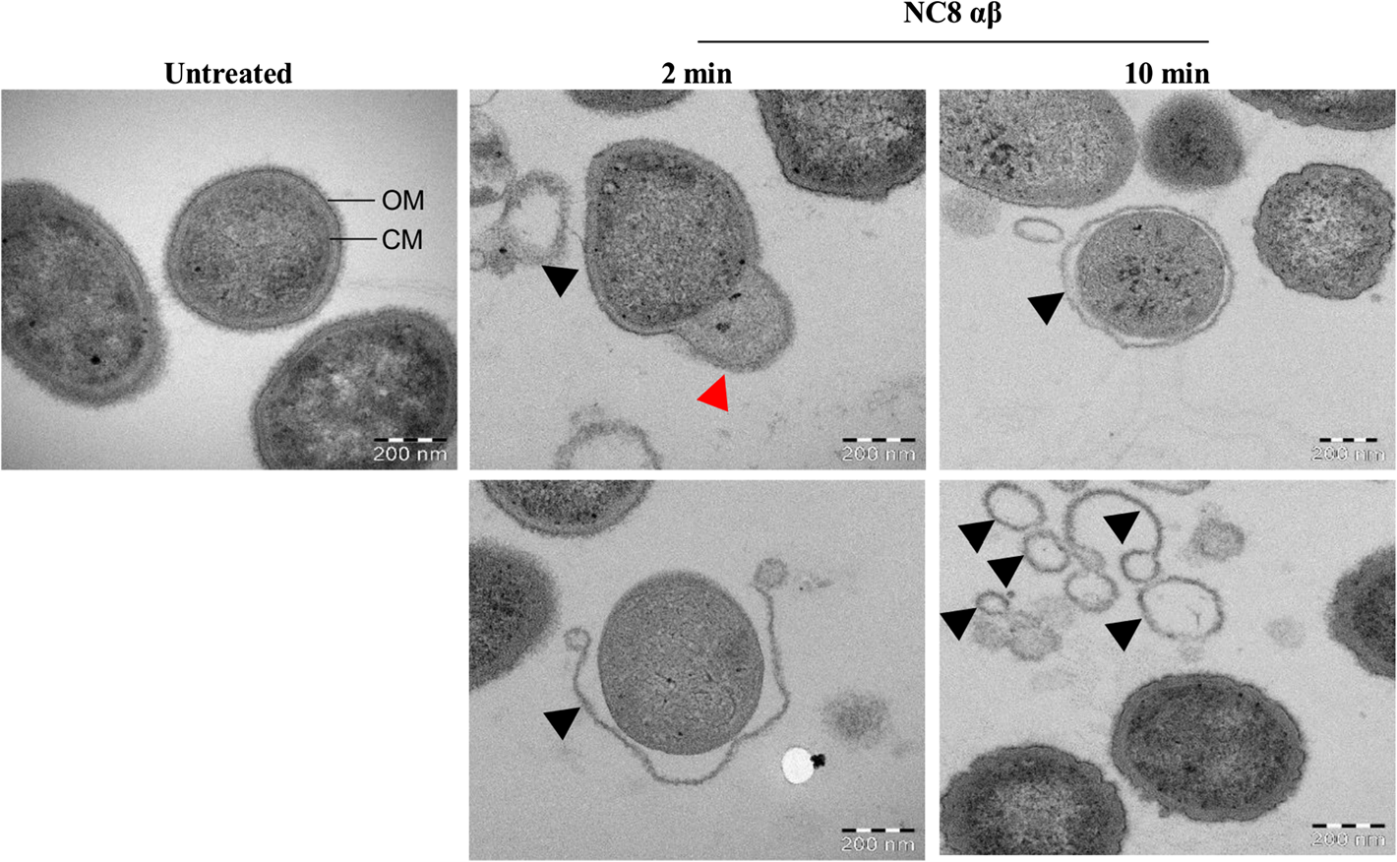
The bacteriocin PLNC8 αβ damages the membrane of *P. gingivalis*. Bacterial ultrastructure was examined using a Hitachi HT 7700 transmission electron microscope and showed typical coccobacillus shapes of untreated *P. gingivalis*, where the outer membrane (OM) and cytoplasmic membrane (CM) could be clearly distinguished. Treatment with PLNC8 αβ (1:2) efficiently damaged *c*cells by causing rupture of the bacterial membrane and leakage of intracellular content (red arrow head), eventually resulting in completely detached outer membrane (black arrow heads). Scale bar = 200 nm

The binding and antimicrobial effect of PLNC8 αβ on *P. gingivalis* is evident, which prompted us to visualize the nature of the damage caused by these peptides. *P. gingivalis* ATCC 33277 were either left untreated or treated with 280 nM of PLNC8 αβ for 2 and 10 min, followed by morphological studies using TEM. Untreated *P. gingivalis* cells showed typical coccobacillus morphology in which the outer and inner membrane are apparent and can be distinguished (Fig. 7). Interestingly, treatment of the bacteria with PLNC8 αβ for 2 min resulted in leakage of intracellular content, which may be due to pore formation. Furthermore, bacteriocin treatment caused detachment of the outer membrane. After 10 min of treatment, a large number of distorted and completely separated membranes were observed. These findings confirm that the effects of PLNC8 αβ on *P. gingivalis* are specific and instant.

## Discussion

The overuse of antibiotics have increased the occurrence of complications in healthcare systems due to bacterial resistance, which in turn have resulted in an intense search for new and effective antimicrobials with less possibility to induce antimicrobial resistance. This study suggests that *Lactobacillus* and bacteriocin PLNC8 αβ can be used as an alternative method to antibiotic therapy in the prevention and treatment of infectious diseases, including periodontitis.

*Lactobacillus* species are part of the normal flora of humans, and have been reported to be important contributors to the balance of oral microflora by preventing pathogen colonization and thus maintaining a healthy state [22]. However, the mechanisms by which *Lactobacillus* interacts with periodontal pathogens and promotes health benefits are sparsely investigated. The oral cavity harbors a wide variety of *Lactobacillus* species, including *L. gasseri, L. salivarius*, *L. brevis, L. plantarum* and *L. rhamnosus* [22, 23]*.* Although the majority of these species are frequently found in the mouth, we show that they possess different specificity towards inhibition of *P. gingivalis*. While *L. brevis* 30670 was ineffective at inhibiting growth of the four *P. gingivalis* strains investigated in this study, both *L. plantarum* NC8 and 44048 were able to significantly suppress *P. gingivalis* growth. Furthermore, although inhibition zones correlate with the ability of an antimicrobial agent to diffuse through a semi-solid matrix, the Kgp-deficient *P. gingivalis* strain K1A was more susceptible to growth inhibition compared to its parent wild type strain W50 and Rgp-deficient strain E8, indicating an essential role for Kgp in *P. gingivalis* survival. The inhibition may possibly be due to secretion of active antimicrobial peptides, as the cell-free conditioning media from *L. plantarum* NC8 and 44048, but not *L. brevis* 30670, caused obvious inhibition, independent of the low pH.

*L. plantarum* NC8 has previously been reported to express a two-peptide bacteriocin, composed of PLNC8 α and β, in the presence of other bacteria [24, 25]. We show that these peptides are effective against *P. gingivalis* by damaging the membrane integrity and allowing the fluorescent dye Sytox Green to enter. By considering the amino acid sequences of PLNC8 αβ (Additional file 1: Figure S1), it is evident that lysine residues (PLNC8 α-4; PLNC8 β-5) are more abundant than arginine residues (PLNC8 α-1; PLNC8 β-1). It is therefore reasonable to suggest a role for the proteinase Kgp in cleaving PLNC8 α and β. This is supported by the fact that *P. gingivalis* lacking Kgp was more susceptible to growth inhibition by *L. plantarum* NC8, compared to WT W50. The proteinases Kgp and RgpA/B account for >80 % of the proteolytic activity of *P. gingivalis* [26]. Studies have established the potent enzymatic activity of cysteine proteinases through degradation of cytokines [27], epithelial junctional proteins [28] and immune receptors [29, 30]. We have recently shown that Kgp, but not Rgps, was associated with suppression of CXCL8 and IL-6 accumulation, induction of TGF-β1 and fibroblast cell viability [31]. The possible degradation of PLNC8 α and β by *P. gingivalis*-derived proteinases is currently under investigation.

Although bacteriocins are generally considered to target bacteria that are closely related to the bacteriocin-producing species, our results suggest that PLNC8 αβ may be used to suppress *P. gingivalis* growth and prevent its establishment. The reported narrow-spectrum activity of most bacteriocins [2] could suggest that their mechanism of action is primarily mediated through binding to specific receptors. Diep and colleagues [32] showed that the class II bacteriocin lactococcin A from *Lactococcus lactis*, binds to the membrane-located components IIC and IID of the mannose phosphotransferase system on target bacteria. Although *P. gingivalis* contains functional glucose/galactose transporters, these processes have mainly been associated with the LPS synthesis pathway [33]. Genetic analysis of *P. gingivalis* suggest that amino acids are the primary source for growth, while the uptake/metabolism of carbohydrates is limited [34]. Furthermore, plantaricin EF (two-peptide bacteriocin, subclass IIb) from *L. plantarum* C11 did not exploit the mannose phosphotransferase system to kill susceptible bacteria [32], suggesting that the two-peptide bacteriocins have a different mechanism of action. The calculated net charge for PLNC8 α and β at pH 7 is 4.1 and 5.2, respectively. These positively charged peptides may be attracted to the negatively charged components lipid A and phosphate groups of the LPS molecule, which initiates the initial interaction prior to pore formation. By incorporating LPS or lipid A into liposomes, to resemble the outer membrane of gram-negative bacteria, Matsuzaki et al. [35] were able to show that the antimicrobial peptide magainin was attracted to the negatively charged lipid A and formed a helix upon binding. This appears to be a common mechanism applied by a range of different naturally occurring and synthetic cationic antimicrobial peptides [36–38], and could thus be used to reduce LPS-induced inflammation and sepsis. Scott and colleagues [39] showed that neutralization of the inflammatory effects of LPS by human α-defensin-1, human β-defensin-2 and other synthetic peptides, was due to prevention of LPS interaction with the acute-phase LPS-binding protein.

Our studies with model membranes suggest that the bacteriocins may interact with lipid membranes without a specific target epitope. The interaction is most likely due to an initial electrostatic attraction between the cationic bacteriocins (Additional file 1: Figure S1) and the anionic lipid membrane, which is commonly seen in the mechanisms of many antimicrobial peptides [40]. It has previously been shown that *P. gingivalis* cells are highly negatively charged at pH > 3 [41]. An increased concentration of bacteriocins on the liposome surface will in turn cause an increase in permeability of the liposomes. This increased permeability is related to the structural changes that were observed when the bacteriocins interact with the liposome surface, likely due to partition-folding coupling due to their amphipathic properties (Additional file 1: Figure S1) [40]. The studies on the model membrane showed that both bacteriocins individually cause disruption of the membrane integrity, although the effect was more pronounced for PLNC8 β.

Efforts have been made to screen for and synthesize antimicrobial peptides against *P. gingivalis*, considering its role as a keystone pathogen in periodontitis. In a study where 236 lactic acid bacterial isolates from food were included, none of the tested strains was able to affect the growth of gram-negative periodontal pathogens, including *P. gingivalis* [42]. Antimicrobial agents from *L. paracasei* HL32 have been shown to suppress *P. gingivalis* growth [43, 44], and a short biosynthetic peptide, Pep-7, was produced and demonstrated to be selectively active against *P. gingivalis* [45]. We show that the antimicrobial effect of the two-peptide bacteriocin PLNC8 αβ occur rapidly and is very efficient, using liposomes and viable *P. gingivalis*.

## Conclusions

In this study, we show that *L. plantarum* NC8 and 44048 are able to suppress *P. gingivalis* growth. Furthermore, bacteriocin PLNC8 αβ from *L. plantarum* NC8 are able to bind, at the nanomolar range, to *P. gingivalis* and cause cellular distortion through detachment of the outer membrane and bacterial lysis. Soluble or immobilized PLNC8 αβ bacteriocins may be used to prevent *P. gingivalis* colonization and pathogenicity, and thus supplement the host immune system against invading pathogens associated with periodontitis and other systemic inflammatory diseases, including atherosclerosis.

## Additional file

Additional file 1: Figure S1. Hydropathy scores of PLNC8 α and β. The amino acid sequence of PLNC8 α (A) and PLNC8 β (B) and their corresponding hydropathy score [46]. (TIF 369 kb)

## Abbreviations

CF: 5 (6)-carboxyfluorescein
EDC: N-ethyl-N0-(3 diethylaminopropyl) carbodimide
Kgp: Lysine gingipain
KRG: Krebs-ringer glucose buffer
NHS: N-hydroxysuccinimide
POPC: 1-palmitoyl-2-oleoyl-sn-glycero-3-phosphatidylcholine
POPS: 1-palmitoyl-2-oleoyl-sn-glycero-3-phospho-L-serine
Rgp: Arginine gingipain
SPR: Surface plasmon resonance
TEM: Transmission electron microscopy
