# Supplementary figures and images for: Antibacterial effects of Lactobacillus and bacteriocin PLNC8 αβ on the periodontal pathogen Porphyromonas gingivalis
Source: BMC Microbiol. 2016 Aug 18;16:188. doi: 10.1186/s12866-016-0810-8 (PMC4990846; doi:10.1186/s12866-016-0810-8)

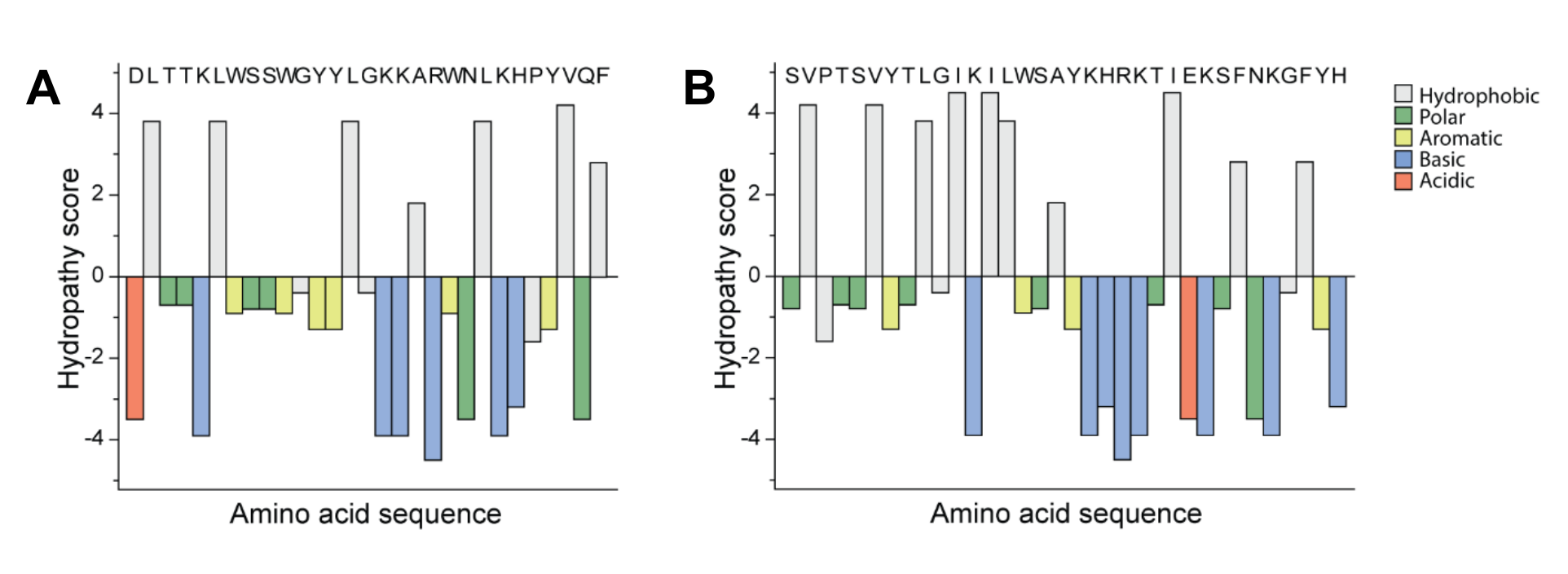

Supplement: Additional file 1: Figure S1. — Hydropathy scores of PLNC8 α and β. The amino acid sequence of PLNC8 α (A) and PLNC8 β (B) and their corresponding hydropathy score [46]. (TIF 369 kb) [file 12866_2016_810_MOESM1_ESM.tif]
